# Neuroanatomical correlates of prion disease progression - a 3T longitudinal voxel-based morphometry study

**DOI:** 10.1016/j.nicl.2016.10.021

**Published:** 2016-11-02

**Authors:** Enrico De Vita, Gerard R Ridgway, Mark J White, Marie-Claire Porter, Diana Caine, Peter Rudge, John Collinge, Tarek A Yousry, Hans Rolf Jager, Simon Mead, John S Thornton, Harpreet Hyare

**Affiliations:** a Lysholm Department of Neuroradiology, National Hospital for Neurology and Neurosurgery, UCLH Hospitals NHS Foundation Trust, Box 65, Queen Square, London WC1N 3BG, United Kingdom; b Neuroradiological Academic Unit, Department of Brain Repair and Rehabilitation, UCL Institute of Neurology, Queen Square, London WC1N 3BG, United Kingdom; c Wellcome Trust Centre for Neuroimaging, UCL Institute of Neurology, 12 Queen Square, London WC1N 3BG, United Kingdom; d FMRIB Centre, Nuffield Department of Clinical Neurosciences, University of Oxford, John Radcliffe Hospital, Oxford, OX3 9DU, United Kingdom; e National Prion Clinic, National Hospital for Neurology and Neurosurgery, UCLH Hospitals NHS Foundation Trust, Box 98, Queen Square, London WC1N 3BG, United Kingdom; f MRC Prion Unit, Department of Neurodegenerative Diseases, UCL Institute of Neurology, Queen Square House, Queen Square, London WC1N 3BG, United Kingdom

**Keywords:** Prion disease, Structural MRI, Longitudinal voxel based morphometry, 3T MRI, CJD

## Abstract

**Purpose:**

MRI has become an essential tool for prion disease diagnosis. However there exist only a few serial MRI studies of prion patients, and these mostly used whole brain summary measures or region of interest based approaches. We present here the first longitudinal voxel-based morphometry (VBM) study in prion disease. The aim of this study was to systematically characterise progressive atrophy in patients with prion disease and identify whether atrophy in specific brain structures correlates with clinical assessment.

**Methods:**

Twenty-four prion disease patients with early stage disease (3 sporadic, 2 iatrogenic, 1 variant and 18 inherited CJD) and 25 controls were examined at 3T with a T1-weighted 3D MPRAGE sequence at multiple time-points (2–6 examinations per subject, interval range 0.1–3.2 years). Longitudinal VBM provided intra-subject and inter-subject image alignment, allowing voxel-wise comparison of progressive structural change. Clinical disease progression was assessed using the MRC Prion Disease Rating Scale. Firstly, in patients, we determined the brain regions where grey and white matter volume change between baseline and final examination correlated with the corresponding change in MRC Scale score. Secondly, in the 21/24 patients with interscan interval longer than 3 months, we identified regions where annualised rates of regional volume change in patients were different from rates in age-matched controls. Given the heterogeneity of the cohort, the regions identified reflect the common features of the different prion sub-types studied.

**Results:**

In the patients there were multiple regions where volume loss significantly correlated with decreased MRC scale, partially overlapping with anatomical regions where yearly rates of volume loss were significantly greater than controls. The key anatomical areas involved included: the basal ganglia and thalamus, pons and medulla, the hippocampal formation and the superior parietal lobules. There were no areas demonstrating volume loss significantly higher in controls than patients or negative correlation between volume and MRC Scale score.

**Conclusions:**

Using 3T MRI and longitudinal VBM we have identified key anatomical regions of progressive volume loss which correlate with an established clinical disease severity index and are relevant to clinical deterioration. Localisation of the regions of progressive brain atrophy correlating most strongly with clinical decline may help to provide more targeted imaging endpoints for future clinical trials.

## Highlights

• Longitudinal brain structural 3T MRI voxel based morphometry in 24 prion disease patients
• In several regions volume loss correlated with increased disease severity as evidenced by decreased prion MRC Scale score
• Prion patients' yearly rates of volume loss are significantly greater than those of healthy controls

## 1 Introduction

Human prion diseases are rare progressive neurodegenerative disorders caused by the propagation of an abnormally folded form of prion protein in the brain (Prusiner, 1982, Collinge, 2000). Three etiological groups include inherited forms caused by mutation of the gene encoding prion protein (inherited), acquired forms from prion contamination of food, medical products or instruments (variant or iatrogenic), and sporadic forms of unknown etiology (Collinge, 2001). Prion diseases are heterogeneous, may mimic other neurodegenerative diseases and may vary in clinical duration from a few weeks to many years. Whilst therapeutic agents that have shown potential to slow disease progression are in preclinical development, there is currently no effective clinical treatment and prion diseases are uniformly fatal.

MRI has become increasingly important for the diagnosis of specific forms of prion disease (Wang et al., 2013). On conventional MRI, classical radiological signs of sporadic CJD are high signal in the striatum on T2-weighted, FLAIR and diffusion-weighted (DW) images, as well as localised cortical or thalamic high signal in FLAIR and DW images (Vitali et al., 2011, Tschampa et al., 2007, Eisenmenger et al., 2016). Abnormally high signal in the pulvinar nucleus of the thalamus compared to anterior putamen bilaterally is a highly specific characteristic of variant CJD (Zeidler et al., 2000).

Quantitative MRI is extremely useful in providing objective data, independent of subjective signal intensity assessments, A number of publications have pioneered quantitative brain MRI in prion diseases, focused in particular upon DW imaging and tissue-volume assessment (Lin et al., 2006, Lee et al., 2009, Cohen et al., 2009 (cerebellar atrophy); Lee et al., 2010, Hyare et al., 2010a, Hyare et al., 2010b, Alner et al., 2012 (cortical thickness); Lee et al., 2012, De Vita et al., 2013 (some VBM atrophy findings); Wang et al., 2013, Caverzasi et al., 2014a, Caverzasi et al., 2014b, Caverzasi et al., 2014a) as well as magnetisation transfer ratio (Siddique et al., 2010) and metabolite concentrations (Galanaud et al., 2010).

Many of these studies have relied upon manually traced regions of interest (ROIs) for numerical analysis. This approach has high anatomical specificity yielding high statistical sensitivity, but is labour intensive and operator dependent, susceptible to intra- and inter-observer variations, and moreover probes only *a priori* selected brain regions. Voxel based morphometry (VBM) (Wright et al., 1995, Ashburner and Friston, 2000) allows voxel-by-voxel MRI group data comparison whereby local, tissue-type specific structural differences can be detected using mass-univariate statistics similar to those employed in fMRI studies. One of the key benefits is the ability to assess all brain regions simultaneously without *a priori* assumptions.

Though atrophy is typically not an early finding on conventional MRI in prion diseases, VBM of cross-sectional MRI has been able to reveal sporadic CJD cerebellar atrophy (Cohen et al., 2009) and, with a more sophisticated normalization procedure (DARTEL (Ashburner, 2007)), patterns of atrophy in the 6-OPRI variant of inherited prion disease (De Vita et al., 2013). Voxel based analysis of DW imaging or diffusion tensor imaging (DTI), using a similar approach to VBM have also been reported (Lee et al., 2009, De Vita et al., 2013). Tract based spatial statistics (Smith et al., 2006), developed to resolve some of the issues associated with white matter tract co-registration has also been used to assess WM disruption in prion disease using DTI (Lee et al., 2012).

An alternative approach to VBM for determining regional volume loss, applied in a prion disease patient cohort by (Caverzasi et al., 2014a, Caverzasi et al., 2014b) automatically parcellates a large number, *i.e.* 40 per hemisphere, of anatomical regions of interest (ROI) using matching algorithms to transfer cortical and subcortical ROI labels from a manually labelled template to individual structural scans, and is exemplified in the FreeSurfer package (Fischl et al., 2004).

If quantitative MRI is to be useful in assessing the efficacy of new prion disease treatments, it is important to understand how the presumed progression of brain microstructural changes (formation of prion protein aggregates, spongiosis, astrocytic gliosis, nerve cell loss) influences MRI-derived metrics. Establishing the relationship of these metrics with clinical disease severity over time is an important step in the development of MRI biomarkers for therapeutic trials.

To date only a few publications have investigated serial brain MRI in prion disease patients. Many of these are single case reports (Fox et al., 1997, Kono et al., 2011, Matoba et al., 2001, Tribl et al., 2002), or studies with fewer than ten subjects (Murata et al., 2002) (*n* = 8), (Caverzasi et al., 2014a, Caverzasi et al., 2014b, Eisenmenger et al., 2016) (*n* = 7), (Ukisu et al., 2005) (*n* = 9), whilst Siddique et al. (2010)) studied 18 inherited prion patients.

While earlier MRI studies in prion disease have been performed mainly at 1.5T, contemporary 3T scanners with multi-element receive coils offer significant sensitivity benefits. As part of the UK National Prion Monitoring Cohort study (Thompson et al., 2013), in this work we examined serially with 3T high-resolution brain structural MRI a cohort of patients with various forms of prion disease, the majority being diagnosed with inherited prion disease. We aimed to use longitudinal VBM to objectively characterise progressive structural brain changes without *a priori* assumptions. The longitudinal VBM approach maximizes sensitivity to individual serial changes (Chételat et al., 2005) and has not been used previously to study prion diseases. We hypothesized that measurable macroscopic changes in brain tissue volumes reflect the development of microscopic brain pathology. Measuring local brain-tissue volume changes between first and last scan in patients, we identified regions in which longitudinal tissue volume loss correlated with the concomitant change in the recently proposed MRC Prion Disease Rating Scale (MRC Scale), a functionally-orientated outcome measure for prion disease patients (Thompson et al., 2013). We also computed the yearly rate of tissue volume change in order to map the regional distribution of longitudinal brain atrophy for prion patients in comparison with age-matched healthy individuals. By including a clinically and etiologically heterogenous group of patients in our cohort we highlight key anatomical regions showing atrophy on average across the different forms of prion disease studied.

## 2 Methods

### 2.1 Subjects

This study was approved by the Scotland A Research Ethics Committee and all participating subjects or their carers provided informed consent. From a total cohort of 95 patients undergoing research MRI between September 2009 and March 2013, we included data from all the subjects in which 2 or more artefact-free structural scans were obtained (*n* = 24). Data from 25 healthy control subjects matched for age to the patients and scanned over the same period was also included. A total of 126 scans were analysed. Healthy controls were scanned at 12 monthly intervals whilst symptomatic patients were scanned at 3–6 monthly intervals and rapidly progressive patients at 6–8 week intervals where possible.

All patients had a neurologist-confirmed diagnosis of probable or definite prion disease. Three had the sporadic form (sporadic CJD), 3 the acquired form (1 variant, 2 iatrogenic) and 18 the inherited form (6 P102L, 3 5-OPRI, 3 A117V, 2 6-OPRI, 2 Y163X, one each of D178N and E200K mutations of the prion protein gene). Data from all of the 24 patients were included in the analysis correlating changes in local brain volume and MRC Scale change. Three patients (1 sporadic, 1 iatrogenic, 1 variant) were excluded from the analysis of yearly rates of GM and WM volume change because of an insufficient time interval (< 3 months) between first and last examination (further details below).

### 2.2 MRI

MRI was performed at 3T (TIM Trio; Siemens, Erlangen, Germany) with the conventional body coil for transmission and a 32-channel head-only receive coil. Structural imaging used a 3D T1-weighted MPRAGE sequence with repetition time 2.2 s, echo time 2.9 ms, inversion time 900 ms, echo spacing 6.7 ms, flip angle 10°, Field of View 282 × 282 × 228 mm^3^, matrix 256 × 256 × 208 with 1.1 mm sagittal partitions, acquisition time 9′23″.

### 2.3 Clinical assessment

All participants underwent detailed systematic neurological and neuropsychological examination and scoring on the MRC Scale (Thompson et al., 2013). The MRC Scale examination assesses domains of cognitive function, speech, mobility, personal care/feeding and continence, weighted according to their relative importance in the disease, and gives a global indication of disease severity. This 20-point functionally-oriented scale is advantageous over single scales for its simplicity of administration and ability to capture the rapid changes that can characterise the disease. Its performance in a large cohort of sporadic CJD patients demonstrated conformity with the Rasch model, indicating linearity, that is, a decline from 20 to 19 is equivalent in magnitude to a decline from 1 to 0, permitting the valid correlation of group MRC Scale with imaging parameters. This assessment was repeated for each patient on the same day as each of their MRI scans.

### 2.4 Longitudinal VBM preprocessing

Longitudinal VBM was performed using MATLAB with in-house processing scripts and SPM8^1^ tools similarly to methods described in (Rohrer et al., 2013). In brief, the following steps were performed:

(a) Images acquired at several time-points from each subject were first segmented using ‘unified segmentation’ (Ashburner and Friston, 2005) (‘New Segment’ tool), and images were bias corrected and intensity normalised to have consistent white matter (WM) mean intensity.
(b) Images were rigidly aligned then non-rigidly warped to their (iteratively evolving) within-subject average using the high dimensional warping algorithm (HDW) (Ashburner and Friston, 2000). This spatial processing methodology removes the bias that would be present if simply registering to the 1st or last scan (Reuter et al., 2012).
(c) Each of the within-subject averages was then re-segmented as in (a), and individual pseudo-time-points were obtained by multiplying the average tissue segment by the Jacobian of the deformation needed to warp the original dataset to the within-subject average, similarly to (Chételat et al., 2005). This *modulation* accounts for warp-associated volume changes (Mechelli et al., 2005). For each subject, the total intracranial volume (TIV) was calculated from each within-subject average by adding up GM, WM and CSF tissue segments volumes.
(d) DARTEL (Ashburner, 2007) was used for the 49 individual subject averages to generate cohort-specific GM and WM templates at (1.5 mm)(Ashburner and Friston, 2005) resolution.
(e) Individual subject GM and WM segments from (d) were warped to these template voxel intensities again modulated to account for normalization-associated volume changes (Mechelli et al., 2005). Smoothing was then performed (6 mm full width half maximum Gaussian kernel).
(f) The ‘optimal threshold’ method (Ridgway et al., 2009) was applied separately to the average GM and WM segments to generate inclusion masks for statistical analysis.

### 2.5 Statistical analysis

Differences between age at 1st scan and TIV between groups were assessed with a *t*-test.

#### 2.5.1 Assessment of regional correlations between brain volume loss and MRC Scale

The MRC Scale captures a number of clinical features of prion disease that appear to be most valuable in assessing disease progression (Thompson et al., 2013). To determine the validity of longitudinal VBM as a marker of clinically meaningful disease progression, we aimed to identify neuroanatomically specific regions in which volume loss correlated with change in individual MRC Scale over the same period in this patient group.

The cohort we studied was clinically and etiologically heterogenous and comprised patients demonstrating both slow and rapid deterioration of functional and neurological skills, reflected in markedly variable rates of decline in MRC Scale with time, with trajectories that are not necessarily linear over the whole monitoring period, especially in the initial and final stages (Thompson et al., 2013). As a consequence, it was not appropriate in group analyses to assess imaging changes over a common time interval for all patients, and therefore we correlated group regional brain-volume change with MRC Scale decline, each variable computed as the difference between baseline and final examination values.

Subjects for whom the maximum inter-scan interval was very small have problematically imprecise rates of change. Thus, for the purpose of establishing associations between longitudinal tissue volume and MRC Scale decline, absolute changes were correlated rather than temporal rates of change. In contrast, working directly with absolute changes ensures data for all patients contribute similarly, whether they are slow-progressing or fast-progressing.

Therefore, for all 24 patients, MRC Scale change (ΔMRC) was calculated as the difference between scores obtained at the times of the first and last MRI examinations. Correspondingly GM and WM volume differences (ΔGM and ΔWM) between first and last MRI scans were computed. Voxel-wise correlation was performed between ΔGM (and ΔWM separately) and ΔMRC in SPM, using the intermediate age of each patient between first and last scan (age_Last_ + age_First_)/2 as a covariate of no interest.

To provide upper and lower bound effect size estimates, we report the average of the slope for the regression of MRC and volume change computed over (a) the significant voxels, which will yield an inflated estimate due to circularity (Kriegeskorte et al., 2010), and (b) all in-mask voxels, which will yield an underestimate due to dilution from unaffected voxels.

For some of the regions that showed significant results, we manually drew ROIs on corresponding anatomical structures on the T1-weighted images and plotted for each of the 24 patients, the variation in GM (ΔGM) *vs* the variation in MRC Scale (ΔMRC) between first and last scan. We also performed a linear regression of ΔGM *vs* ΔMRC.

#### 2.5.2 Assessment of regional yearly GM and WM rates of change

We also assessed the patterns of longitudinal brain atrophy in prion patients compared with controls. Voxel-wise yearly rates of GM and WM volume change, presumed to represent atrophy rates when negative and local growth/swelling rates if positive, were calculated by linear regression using all available individual time-points for each subject.

For this analysis, subjects with maximum inter-scan intervals of < 3 months were excluded, to avoid disproportionate propagation of measurement noise caused by small changes in the short interval, as discussed above. As a consequence data from 1 sporadic, 1 iatrogenic and 1 variant patient were not included in the analysis.

Statistically significant group differences in yearly voxel-wise volume change between controls and the remaining patients (*n* = 21), were evaluated separately for GM and WM, with intermediate age between first and last scan as a covariate of no interest. Effect size maps were also computed showing the difference in yearly volume change between patients and controls.

Here, for illustration of effect size we reported the average effect over all significant voxels (upper bound) as well as over a mask defined by the voxels where the average slope across controls and patients is significant (lower bound). Note that the difference is essentially orthogonal to the average, due to the almost equal group sizes, so there is no concern over circularity with this effect size estimate.

#### 2.5.3 Statistical thresholds and display

SPM-t maps were computed, corrected for multiple comparisons using false discovery rate (FDR) with *p* < 0.05. Although analysis of WM and GM fractional tissue volume differences were performed separately, for brevity results are shown on the same figures with tissue-type indicated by different colours.

SPMs were affine transformed to the standard MNI anatomical space, using parameters obtained by affine-registering the DARTEL template to the MNI space tissue prior-probability maps. Results are displayed overlaid on the average of all warped and smoothed T1 volumes, transformed to MNI space. All are presented using the radiological convention (patient's right hemisphere on left of the figures).

## 3 Results

Summary statistics for the group age at first scan and TIV are given in Table 1, neither was significantly different between controls and patients groups (*p* = 0.7 for both). There was also no difference in gender between groups (*p* = 0.5).

### 3.1 Timing of MRI examinations

Nineteen out of the 49 subjects had more than two scans: 6 controls (with 3 scans), 13 patients (8 with 3 scans, 1 with 4, 2 with 5, 2 with 6 scans). Supplementary Fig. S1 displays individual subject examination timepoints (in years from the initial MRI scan)) for patients (a) and controls (b).

All subjects underwent at least 2 examinations, the second performed at 1.2 ± 0.49 (mean ± SD) years post-baseline for *controls* (range 0.44–2.93 years), and 0.55 ± 0.27 years (range 0.12–1.02 years), for *patients*. For those subject undergoing 3 examinations, the third timepoint was at 1.7 ± 0.41 years for *controls* (*n* = 6), and 1.03 ± 0.64 years (*n* = 16) for *patients*. Including all subjects the interval between first and last scan was 1.38 ± 0.53 years for controls and 1.19 ± 0.91 years for *patients* (no difference, *p* = 0.4).

### 3.2 MRC scale

**Fig. 1:**
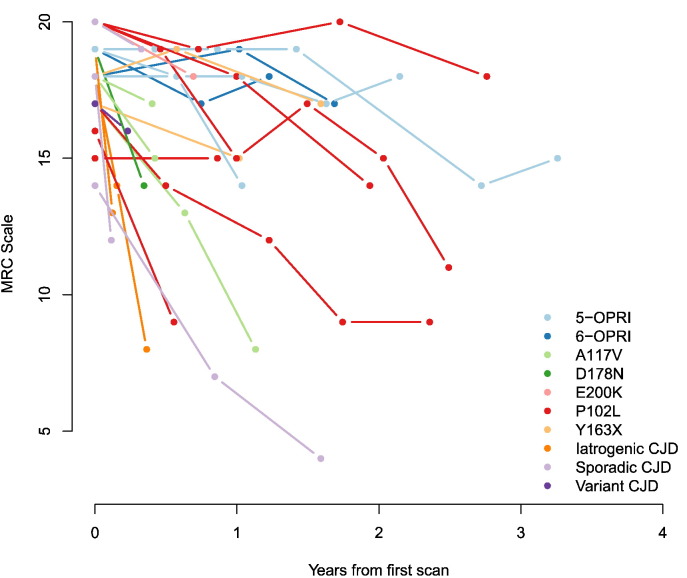
MRC Scale change over time for each of the 24 patients. Different etiologies and genetic mutations are shown in different colours.

All controls had an MRC Scale of 20. The patients entered the trial at an early disease stage: the mean (sd) MRC Scale at first scan for patients was 18.1 (1.6) with median (range) 18 (14–20). As expected, for patients the MRC Scale typically decreased monotonically, with varying rates of decline. The MRC Scale on the last scan was 13.9 (4.0), with a median (range) was 15 (4–19). The MRC Scale trajectories for each of the 24 patients are shown in Fig. 1 with different etiologies or inherited mutations shown in different colours. It is apparent that there is a range of rates of clinical progressions, and even patients with the same genetic mutation or etiology can progress at very different rates.

Table 2 summarises for each patient, the prion disease form (or specific genetic mutation for the inherited cases), age, the disease duration at first scan (date from onset of first symptoms) the total number of scans, the time interval between first and last scan and ΔMRC.

### 3.3 Imaging results

#### 3.3.1 Correlation of brain volume changes with MRC Scale change in patients

**Fig. 2:**
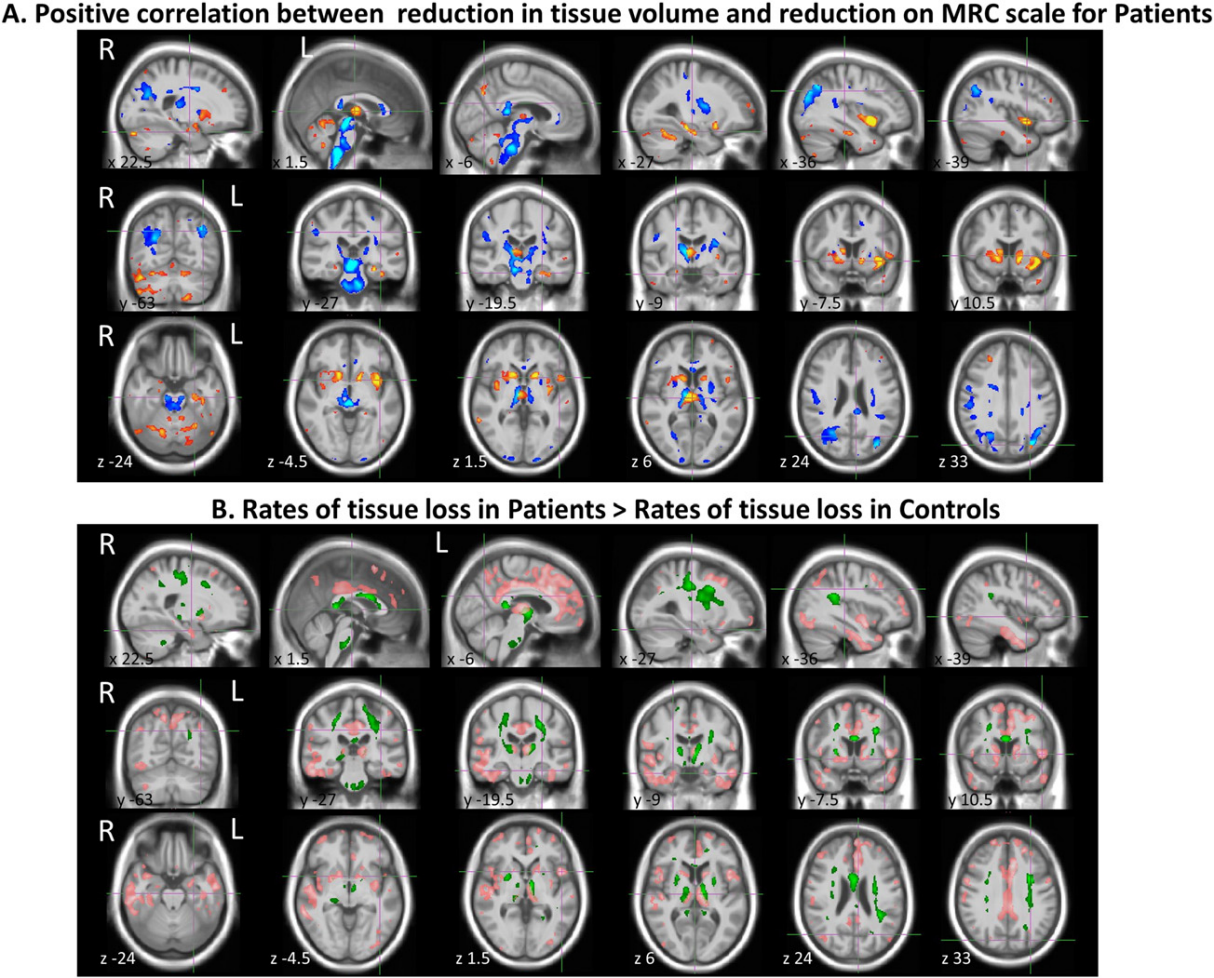
(A) SPM-t maps showing significant positive correlation (FDR *p* < 0.05) between reduction in tissue volume, ΔGM (red-yellow) or ΔWM (blue-cyan), and ΔMRC Scale when comparing first and last scan. For GM there were 10,149 suprathreshold (*t* > 3.31) voxels over 308,090 tested; the median/mean t (sd) over these voxels was 3.75/3.91(0.55); for WM there were 15,636 voxels (with *t* > 2.88) over 176,008; the median/mean t over these was 3.46/3.75(0.85). (B). SPM-t maps showing GM (pink) and WM (green) areas where atrophy in patients was greater than in controls (FDR *p* < 0.05). For GM there were 35,121 supra-threshold (*t* > 2.64) voxels over 308,090 tested; the median/mean t (sd) over these was 3.02/3.12(0.40); for WM: 8740 supra-threshold (*t* > 2.96) over 176,008, with median/mean t (sd) 3.40/3.52(0.46). For each section, MNI coordinates are shown.

There was a significant correlation between tissue volume change between the first and last examination, and change in MRC Scale over the same period, across several brain areas, with decreases in MRC Scale accompanied by decreases in local GM and/or, WM tissue volumes. Considering the whole analysis mask the mean fractional volume decrease in GM (WM) was 0.00181 (0.00227) per each point decrease in MRC Scale. No areas demonstrated a significant negative correlation. Fig. 2A displays the areas of significant positive correlation (at FDR *p* < 0.05) between ΔGM and ΔMRC (red-yellow) as well as between ΔWM and ΔMRC (blue-cyan). In summary, there were clusters showing significant correlation bilaterally in the insular cortex, head of caudate and putamen, thalamus, hippocampus and cerebellum, superior parietal lobule, genu and splenium of the corpus callosum, midbrain, medulla and pons. There were right sided GM clusters in the amygdala, middle temporal gyrus, inferior frontal gyrus and parahippocampaly gyrus. On the left, GM clusters in the precuneus and superior occipital gyrus. Also WM clusters in the vertical fibers of the arcuate fasciculus and thalamo-cortical fibers of the cortico-spinal tract (full results for all significant clusters are reported in Supplementary File 1, Supplementary File 2). In terms of effect size over all significant areas combined, ΔGM between first and last scans were on average 0.018 (0.011) (mean (sd)) and ΔWM 0.029 (0.015); the average slope of ΔGM decrease per one point decrease in MRC Scale was 0.0074(0.0025) for GM and 0.0086(0.0037) for ΔWM.

#### 3.3.2 Yearly rates of GM and WM volume change

The voxel-wise linear rate of tissue volume change for patients was lower (more negative) than for controls in 86.7% of GM tissue voxels and in 71.6% of WM voxels.

**Supplementary Fig. S2:**
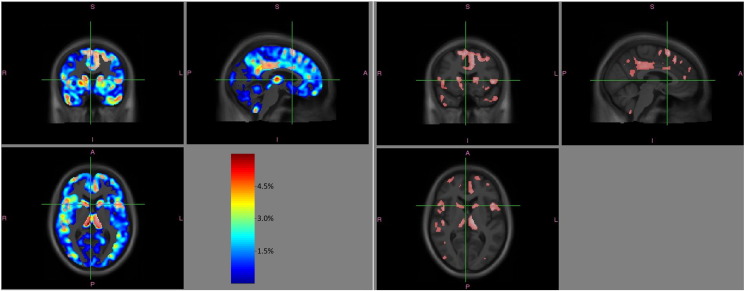
Yearly rate of additional GM fractional volume reduction for patients beyond that seen in controls (left). The colorbar is set to 0–6%. The right-hand-side panel shows for comparison on the same sections the areas of significant differences in yearly rates of GM volume reductions in patients *vs* controls as shown in Fig. 2A (FDR *p* < 0.05).

**Supplementary Fig. S3:**
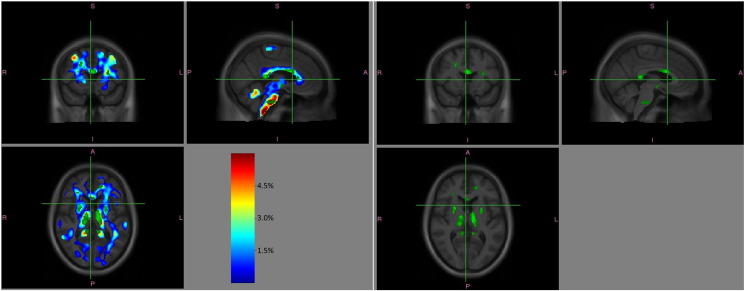
Yearly rate of additional WM fractional volume reduction for patients beyond that seen in controls (left). The colorbar is set to 0–6%. The right-hand-side panel shows for comparison on the same sections the areas of significant differences in yearly rates of WM volume reductions in patients *vs* controls as shown in Fig. 2B (FDR *p* < 0.05).

These differences were significant in numerous anatomical regions (Fig. 2B) (details of significant clusters MNI coordinates in Supplementary File 3, Supplementary File 4). All of these areas were in regions where the patients group displayed on average negative rates of change, thus these findings indicate significant yearly atrophy in patients with respect to controls as confirmed by visual inspection of the effect size maps (for GM Supplementary Fig. S2, for WM Supplementary Fig. S3).

Areas of significantly increased atrophy rates in *patients* that also overlapped with areas showing significant correlation between volume loss and MRC Scale decrease included: insular cortex, hippocampus, head of caudate, thalamus, putamen, the IFG, part of the pons, the genu of the corpus callosum and parts of the corticospinal tract. Other areas with significant atrophy rate differences compared with controls included the body of the corpus callosum, large areas of the cingulate gyrus, parts of the superior and middle temporal gyri, some areas of the superior and middle frontal gyri, and the fusiform gyrus. Supplementary Fig. S4 shows ΔGM *vs* ΔMRC in the putamen (A) and head of caudate (B) in the right hemisphere for each individual patient together with the least square regression line when combining all patients. Patients with different etiologies or inherited mutations are shown in different colours as in Fig. 1.

Over the voxels where the average slope of control and patients was significant (FDR *p* < 0.05), the yearly rate of volume fraction change was − 0.027 (0.011) for patients and 0.0028 (0.0034) for controls for GM; for WM it was 0.0334 (0.0131) for patients and 0.0042 (0.0045) for controls. The corresponding patient-control group differences are 0.0238 (0.0118) for GM and 0.0292 (0.0138) for WM. In terms of average effect size over all significant voxels, the yearly rate of volume fraction change was − 0.0310 (0.0115) for patients and 0.0015 (0.0009) for controls, for GM; for WM it was 0.0377 (0.0138) for patients and 0.0001 (0.0045) for controls. The corresponding patient-control group differences are 0.0325 (0.0122) for GM and 0.0376 (0.0125) for WM.

There were no voxels for which the opposite contrast (rates of WM or GM volume increase in patients significantly greater than in control subjects, or volume decrease in patients slower than in controls) was significant.

## 4 Discussion

We have shown, in patients with various forms of human prion disease and varying individual rates of disease progression, that longitudinal voxel-based morphometry can detect brain regions in which local tissue volume reduction correlates significantly with decline in functional capacity over the same interval. We also quantified the equivalent yearly rate of local tissue volume loss, and determined brain regions in which this was greater than in controls. Combined, these findings support the validity of VBM–determined volume change as a clinically relevant marker of prion disease progression and provide new evidence regarding the neuroanatomical correlates of prion disease.

This is the first study to investigate voxelwise longitudinal volume change in prion disease across the whole brain. Our cohort of 24 patients is one of the largest with this rare condition studied longitudinally, with phenotypic heterogeneity representative of the range of clinical phenotype seen at our national centre. The use of a 32-channel head coil at 3T, is likely to offer improved sensitivity compared with earlier MRI studies in prion disease. Our processing approach, with intra-subject image alignment *via* high-dimensional warping, and inter-subject DARTEL-based warping for longitudinal normalization for VBM, provides accurate normalization of the multiple individual scans and allows voxelwise comparison of longitudinal structural brain changes with modest spatial smoothing (6 mm).

The patient group deteriorated at varying rates, with progressive impairment of cognition, speech, mobility and ability to care for themselves. This is typical of prion disease in general and these different aspects of prion disease progression are reflected in changes in the MRC Scale. Pathologically, this deterioration is due to the hallmark prion disease histopathological changes of progressive accumulation of prion protein aggregates with ensuing spongiosis, astrocytic gliosis and nerve cell loss, common to all aetiological groups.

We hypothesized that these microscopic changes are reflected in measureable WM and GM macroscopic changes. Structural MRI at 1.1 mm isotropic resolution with subsequent 6 mm spatial smoothing, necessarily measures volume changes averaged over a large number of neurons, axons and glial cells. Nevertheless we expect that local tissue integrity, quantified in terms of fractional GM and WM differences estimated by segmentation of T1-weighted images, will be sensitive to these changes. Our ability to detect such changes with statistical significance depends not only on the degree of microscopic tissue disruption, but also on the signal-to-noise-ratio of the measurement.

Prion disease patients displayed significant longitudinal volume loss correlating with decreased MRC Scale, partially overlapping with regions where yearly rates of volume loss significantly exceeded those in controls in a number of areas: head of caudate, putamen, thalamus, hippocampus, insular cortex, inferior frontal gyrus (pars opercularis), splenium and genu of corpus callosum, pons, parts of the superior frontal gyrus, middle temporal gyrus. Although the corpus callosum is non-specific, many of these areas such as the basal ganglia and thalamus are known to show changes on conventional MRI (T2-weighted, FLAIR, DW images) or are functionally associated with specific aspects of prion disease clinical presentation (Tschampa et al., 2007). The putamen and caudate nuclei receive input from diverse cortical areas, including prefrontal and limbic structures with non-motor output from the striatum projecting *via* the mediodorsal and ventrolateral thalamic nuclei to the dorsolateral prefrontal cortex, lateral orbitofrontal cortex and the anterior cingulate and are key structures involved in memory circuits. The mesial temporal lobe and frontal volume loss corresponds well to the known frontal executive dysfunction and memory loss in these patients. Hippocampal involvement has been already observed cross-sectionally in a VBM study of 6-OPRI inherited prion patients performed at 1.5T (De Vita et al., 2013).

The regions where only the correlation between tissue loss and MRC Scale decline reached significance included: parahippocampal gyrus, precuneus, superior parietal lobule, occipital cortex and WM (superior/middle occipital gyrus), parietal WM, vertical fibers of arcuate fasciculus, cortico-spinal tract fibers at the level of the centrum semiovale and the pons. Apraxia is an important feature in prion diseases, known to be associated with parietal lobe dysfunction. Ataxia is known to be associated with pontine pathologies. The correlation between tissue loss and MRC Scale decline appeared to highlight relatively few cortical structures. This may be due to the fact the MRC Scale is a composite rating scale of overall patient function and cannot be directly linked to specific cortical regions.

Significant cortical thinning was shown in the precuneus, inferior parietal cortex, supramarginal gyrus in a group of 18 6-OPRI patients (Alner et al., 2012); VBM demonstrated volume loss in many cortical and WM regions including the corpus callosum in 9 6-OPRI patients (De Vita et al., 2013). VBM was also used to reveal focal cerebellar atrophy in 11 sporadic CJD patients with cerebellar syndrome (Cohen et al., 2009). Cerebellar and widespread supra-tentorial volume loss was also reported in a single P102L gene carrier (Fox et al., 1997). Cortico spinal tract WM involvement was detected by measuring diffusion changes (Lee et al., 2012) (alongside internal capsule, external capsule, fornix, and posterior thalamic radiation). Another study (Caverzasi et al., 2014a) using FreeSurfer reported in sporadic CJD significant cross-sectional changes in diffusion properties in a number of regions, and some cortical thickness reduction trends but could not demonstrate any atrophy, cross-sectionally or longitudinally.

Our results are thus broadly consistent with previous reports on various prion disease sub-types. However these were either cross-sectional or focused on microscopic tissue water distribution changes (*e.g.* DTI), whilst we demonstrated regional macroscopic volumetric changes over time. While it is to be expected that complementary MRI metrics detect change in consistent regions, their temporal relationship – if elucidated in future multimodal longitudinal studies – may provide important pathophysiological insights.

Correlation of local brain volume loss with an established rating scale supports the biological and clinical validity of quantitative MRI as a potential secondary outcome measure in treatment trials. This is particularly relevant since potential therapies aiming to slow prion disease progression are in preclinical development (Freir et al., 2011). While the MRC Scale provides a simple, accessible, yet useful clinically-oriented tool to monitor therapeutic efficacy, quantitative imaging may provide valuable complementary, objective and sensitive measures of progression.

### 4.1 Limitations of the study and future work

Though larger than similar prior studies, our sample size was still limited, due to the rarity of the disease, and the difficulties some patients have with tolerating MRI, especially in late-stage disease. Indeed the number of sporadic CJD patients that could undergo structural MRI without sedation or general anaesthetic at more than one time-point was extremely low: their fraction in our cohort was much lower than their incidence in the wider prion patient population (approximately 85%). Similarly, the fraction of inherited prion disease patients was higher in our cohort than the 10–15% fraction in the wider prion patients population; this reflects the higher relative prevalence and longer clinical duration of this subtype.

Therefore, whilst we still included a range of patients with slow, intermediate and rapid functional/neurological decline (Fig.1), caution is required in generalizing our findings, which may be specific to this group, to wider or more-specific sub-populations. Future multi-centre studies may provide access to a larger range of patients, making more etiologically homogeneous subgroup analyses possible.

Furthermore, whilst our cohort is heterogenous, the underlying neuropathological mechanisms are common across prion sub-types; having effectively taken into account differing rates of functional progression, we were thus able to detect significant specific regional involvement, likely to reflect common neuroanatomic correlates of clinical decline across all prion sub-types included. Studying an heterogenous cohort allowed us to increase numbers and ensuing statistical power.

Voxel-wise statistical analyses must address the problem of multiple-comparison. To asses statistical significance we used a 5% voxelwise false discovery rate threshold, similar to studies using comparable approaches (Rohrer et al., 2013). This threshold limits the number of false negatives but is prone to producing false positives. Nevertheless, the absence of significant regions on opposite contrasts and the overall anatomical consistency of the two analyses (and the overall agreement with present knowledge of prion disease neurodegeneration patterns) provide additional confidence in the validity of the results.

One of the main criticisms of VBM focused on its reliance on imperfect image registration (Bookstein, 2001). To address this, the low-dimensional basis-function approach used in early work has here been replaced with a high-dimensional diffeomorphic approach (Ashburner, 2007) that has been shown to perform very well (Klein et al., 2009).

Methods other than longitudinal VBM exist to study atrophy progression, such as Tensor Based Morphometry (Hua et al., 2011) or methods based on FreeSurfer (Dale et al., 1999, Fischl et al., 1999, Reuter et al., 2012) (see for instance (Caverzasi et al., 2014a, Caverzasi et al., 2014b, Landin-Romero et al., 2016, Holland et al., 2012)). Longitudinal TBM and VBM are very closely related, since both rely on the Jacobian determinants from spatial transformations to characterise volume change. However VBM combines the registration-based volume-change information with tissue probability information. This extra information can increase sensitivity in areas of boundary between atrophic grey matter and expanding CSF where naive smoothing of TBM maps can cause partial cancellation. Procedures based on FreeSurfer's parcellation and segmentation have not yet been carefully compared to longitudinal VBM in the literature, and the relative advantages of each methodology might vary by region. One of the benefits of longitudinal VBM is that it is fully automated whilst FreeSurfer might require time-consuming manual interaction for optimal results in challenging regions [*e.g.* https://surfer.nmr.mgh.harvard.edu/fswiki/Edits]). Further work will explore alternative atrophy assessment techniques.

A few previous studies have shown a relationship between involvement of the striatum and shorter disease course (Gao et al., 2015, Kim et al., 2011, Yi et al., 2008). A recent longitudinal study reported at follow-up an increased DWI signal mainly in the striatum (Eisenmenger et al., 2016). As the present study demonstrates significant atrophy in the striatum, it would be extremely interesting to analyse the relationship between DWI and atrophy changes, across the whole brain and particularly in the striatum. This will be addressed in future work.

## 5 Conclusions

We have presented the first 3T MRI longitudinal voxel-based analysis of brain structural change in prion disease in a cohort of patients with various forms of the disease (the majority having inherited prion disease), at an early stage of the disease course. We identified progressive volume loss in basal ganglia, cortical and WM regions that relate to previous cross-sectional imaging findings and to functional deficits associated with disease progression. With the current data, it is not yet possible to establish whether MRI-determined brain volume loss precedes clinical change quantified according to functional scales such as the MRC Scale. Nevertheless, localisation of regions of progressive brain atrophy correlating most strongly with clinical decline may help to determine more effective imaging endpoints for future clinical trials. In this respect it will be important to compare the usefulness of brain atrophy *vs.* other imaging measures, including diffusion weighted imaging metrics.

The following are the supplementary data related to this article.

Supplementary Fig. S1 Distribution of imaging timepoints for each of the 25 control subjects (A) and each of the 24 patients (B). Supplementary Fig. S1

Supplementary Fig. S4 For each of the 24 patients, the variation in GM (ΔGM) is plotted *vs* the variation in MRC Scale (ΔMRC) between first and last scan for the right putamen (A) and the right head of caudate (B). Different etiologies or inherited mutations are shown in different colours as in Fig. 1. Linear regression equations and R(Ashburner and Friston, 2000) are: y = − 0.004719 + 0.006664 x, R(Ashburner and Friston, 2000) = 0.394 for the right putamen and y = − 0.01274 + 0.00782 x, R(Ashburner and Friston, 2000) = 0.342 for the right head of caudate. Supplementary Fig. S4

Supplementary File 1 Simplified raw SPM results table detailing areas of significant correlations between ΔGM and ΔMRC Scale (FDR *p* < 0.05). (Local Maxima within the same cluster have been removed.) Supplementary File 1

Supplementary File 2 Simplified raw SPM results table detailing areas of significant correlations between ΔWM and ΔMRC Scale (FDR *p* < 0.05). (Local Maxima within the same cluster have been removed.) Supplementary File 2

Supplementary File 3 Simplified raw SPM results table detailing areas where GM atrophy in patients was greater than in controls (FDR *p* < 0.05). (Local Maxima within the same cluster have been removed.) Supplementary File 3

Supplementary File 4 Simplified raw SPM results table detailing areas where WM atrophy in patients was greater than in controls (FDR *p* < 0.05). (Local Maxima within the same cluster have been removed.) Supplementary File 4

## Figures and Tables

**Table 1:** Group ages and total intracranial volume. ^#^: median (range).

	Controls (*n* = 25)	Patients (*n* = 24)
Gender	13 males	13 males
Age at 1st examination^#^ (years)	46.5 (23.8–69.4)	49.0 (25.0–76.7)
Total intracranial volume^#^ (litres)	1.50 (1.29–1.83)	1.52 (1.21–1.85)

**Table 2:** Individual data for patients: prion disease etiology, intermediate age between the first and last scan (Age), disease duration (DD), total number of scans (N_scans_), time interval between first and last scan (t_last_ − t_first_), change in MRC Scale between these timepoints (ΔMRC). ^$^: years. ^#^: first scan before onset. ^&^: first 2 scans before onset.

ID	Prion disease etiology	Age^$^	DD^$^	Nscans	t_last_ − t_first_^$^	ΔMRC
P06	5-OPRI	48.99	2.29	6	3.26	4
P12	5-OPRI	52.22	5.71	3	1.03	4
P13	5-OPRI	48.67	6.71	5	2.15	1
P08	6-OPRI	42.51	3.63	3	1.23	1
P05	6-OPRI	42.25	5.85	3	1.69	1
P15	A117V	38.50	1.66	2	0.40	1
P16	A117V	51.21	2.18	2	0.42	3
P03	A117V	38.57	1.21	3	1.13	9
P09	D178N	52.67	0.63	2	0.35	5
P20	E200K	46.19	-0.50^#^	2	0.69	2
P04	P102L	50.73	2.52	2	0.86	0
P07	P102L	62.98	1.57	5	2.36	8
P17	P102L	56.48	-0.77^&^	4	2.76	2
P19	P102L	55.23	1.38	2	0.56	7
P11	P102L	61.07	0.87	3	1.94	6
P14	P102L	42.85	0.04	6	2.49	9
P18	Y163X	43.72	9.98	3	1.59	1
P10	Y163X	54.19	15.23	2	1.02	2
P01	Iatrogenic CJD	47.08	0.43	3	0.36	10
P02	Iatrogenic CJD	44.15	0.75	2	0.12	6
P21	Sporadic CJD	59.36	0.62	2	0.12	6
P22	Sporadic CJD	59.50	2.21	3	1.59	10
*P*23	Sporadic CJD	77.00	0.96	2	0.33	1
P24	Variant CJD	25.12	0.22	2	0.23	1

## Footnotes

1 http://www.fil.ion.ucl.ac.uk/spm/software/spm8/.
