# Supplementary figures and images for: Neuroanatomical correlates of prion disease progression - a 3T longitudinal voxel-based morphometry study
Source: Neuroimage Clin. 2016 Nov 2;13:89–96. doi: 10.1016/j.nicl.2016.10.021 (PMC5133666; doi:10.1016/j.nicl.2016.10.021)

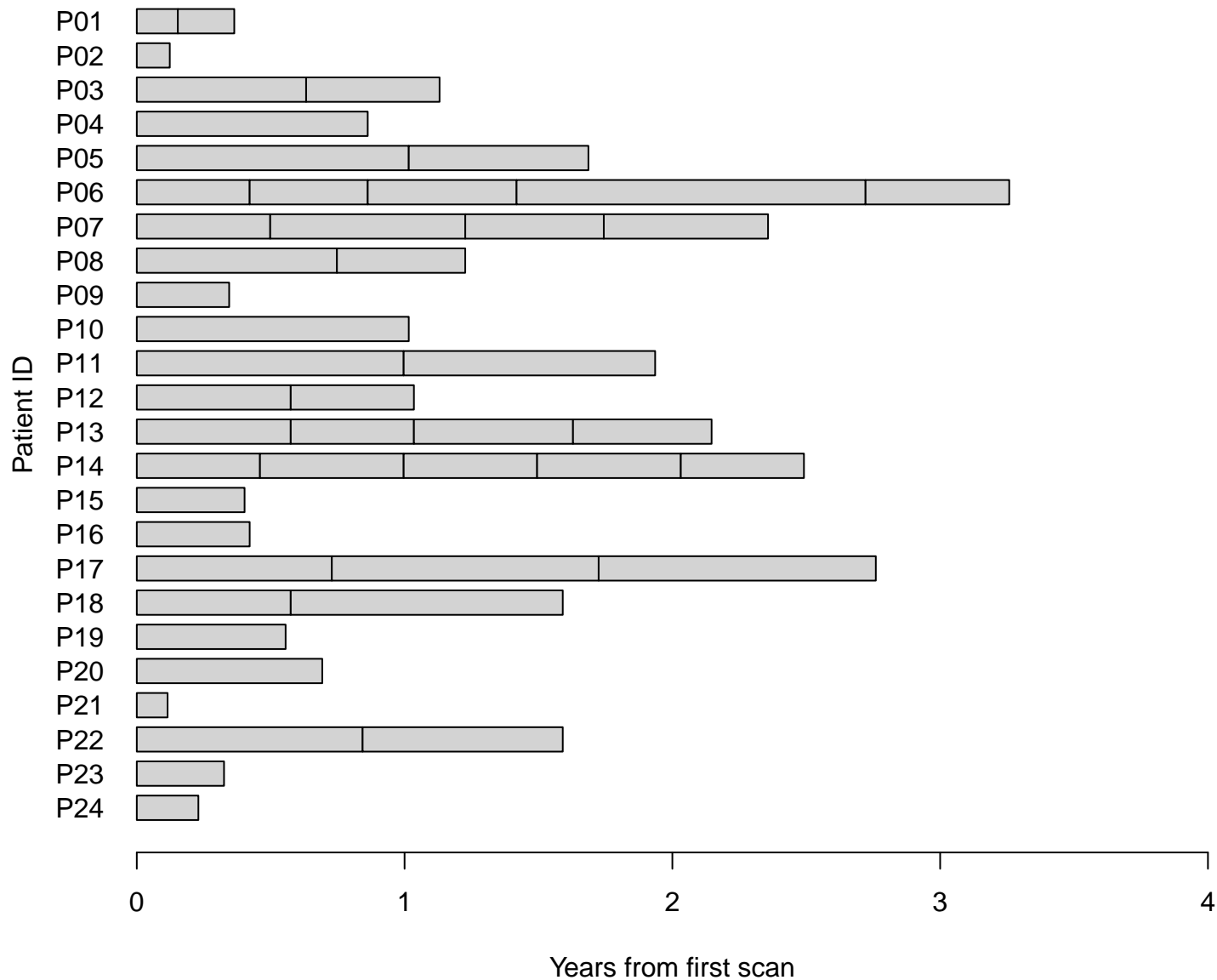

Supplement: Supplementary Fig. S1 — Distribution of imaging timepoints for each of the 25 control subjects (A) and each of the 24 patients (B). [file mmc1.pdf]

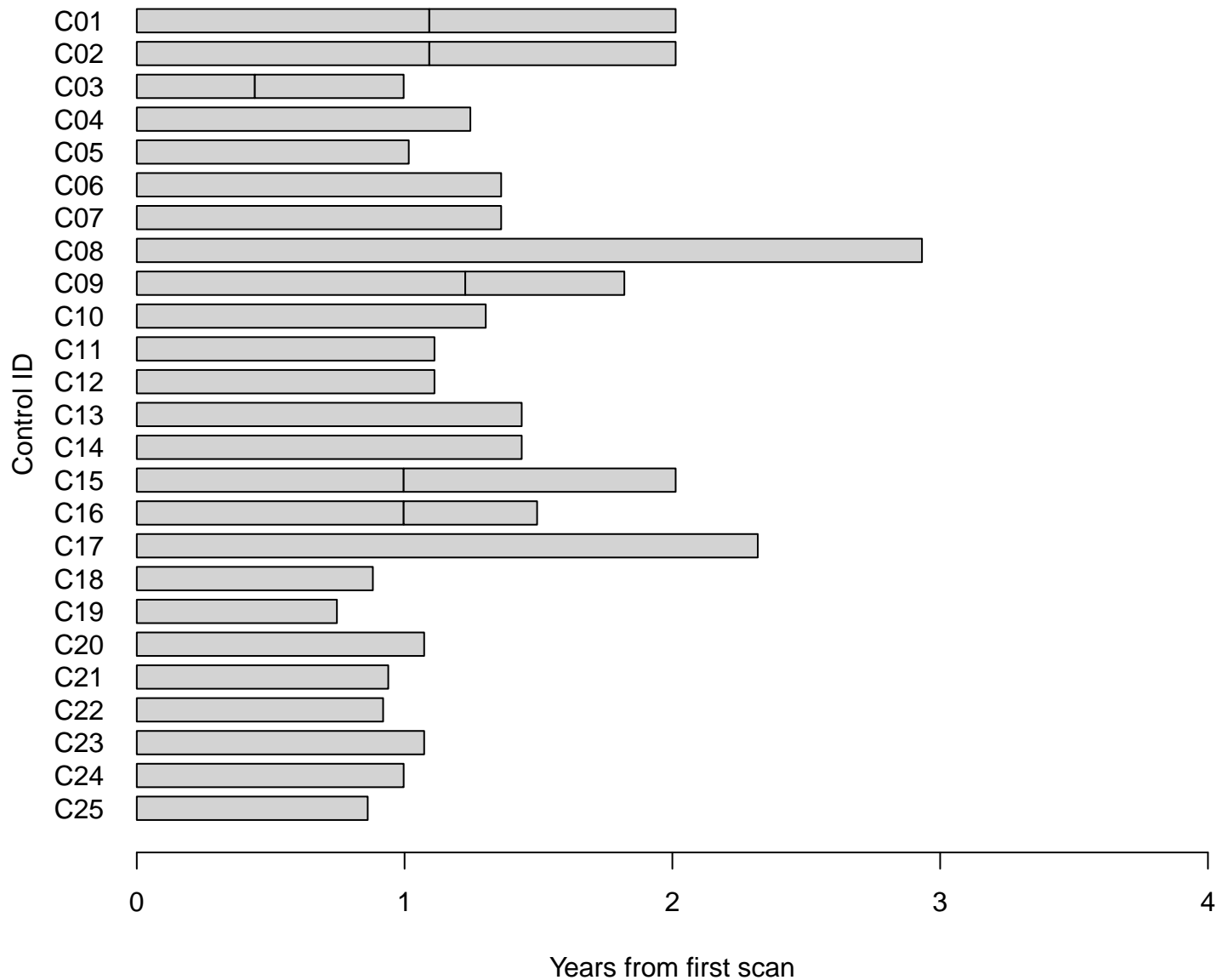

Supplement: Supplementary Fig. S1 — Distribution of imaging timepoints for each of the 25 control subjects (A) and each of the 24 patients (B). [file mmc2.pdf]

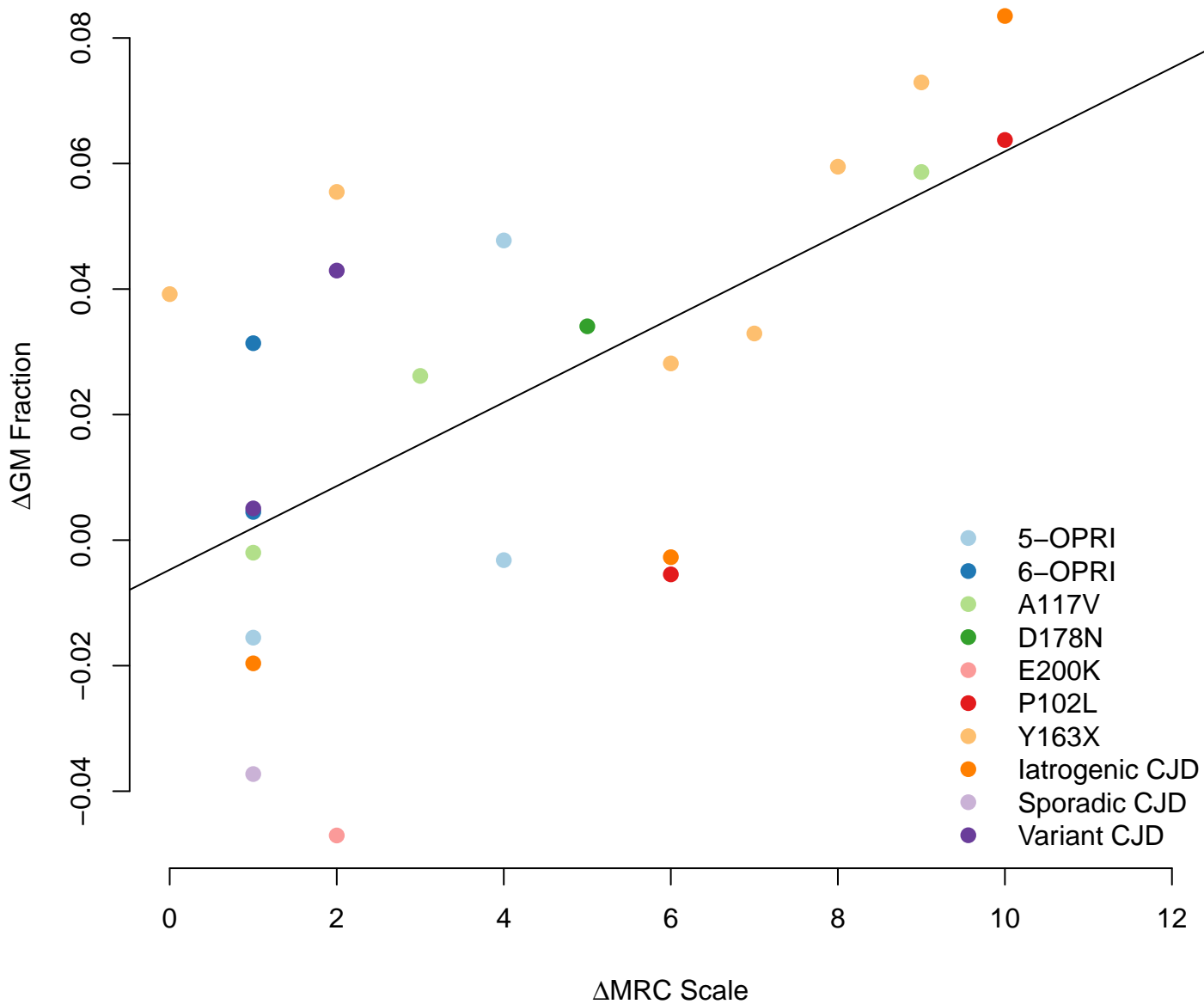

Supplement: Supplementary Fig. S4 — For each of the 24 patients, the variation in GM (ΔGM) is plotted vs the variation in MRC Scale (ΔMRC) between first and last scan for the right putamen (A) and the right head of caudate (B). Different etiologies or inherited mutations are shown in different colours as in Fig. 1. Linear regression equations and R(Ashburner and Friston, 2000) are: y = − 0.004719 + 0.006664 x, R(Ashburner and Friston, 2000) = 0.394 for the right putamen and y = − 0.01274 + 0.00782 x, R(Ashburner and Friston, 2000) = 0.342 for the right head of caudate. [file mmc3.pdf]

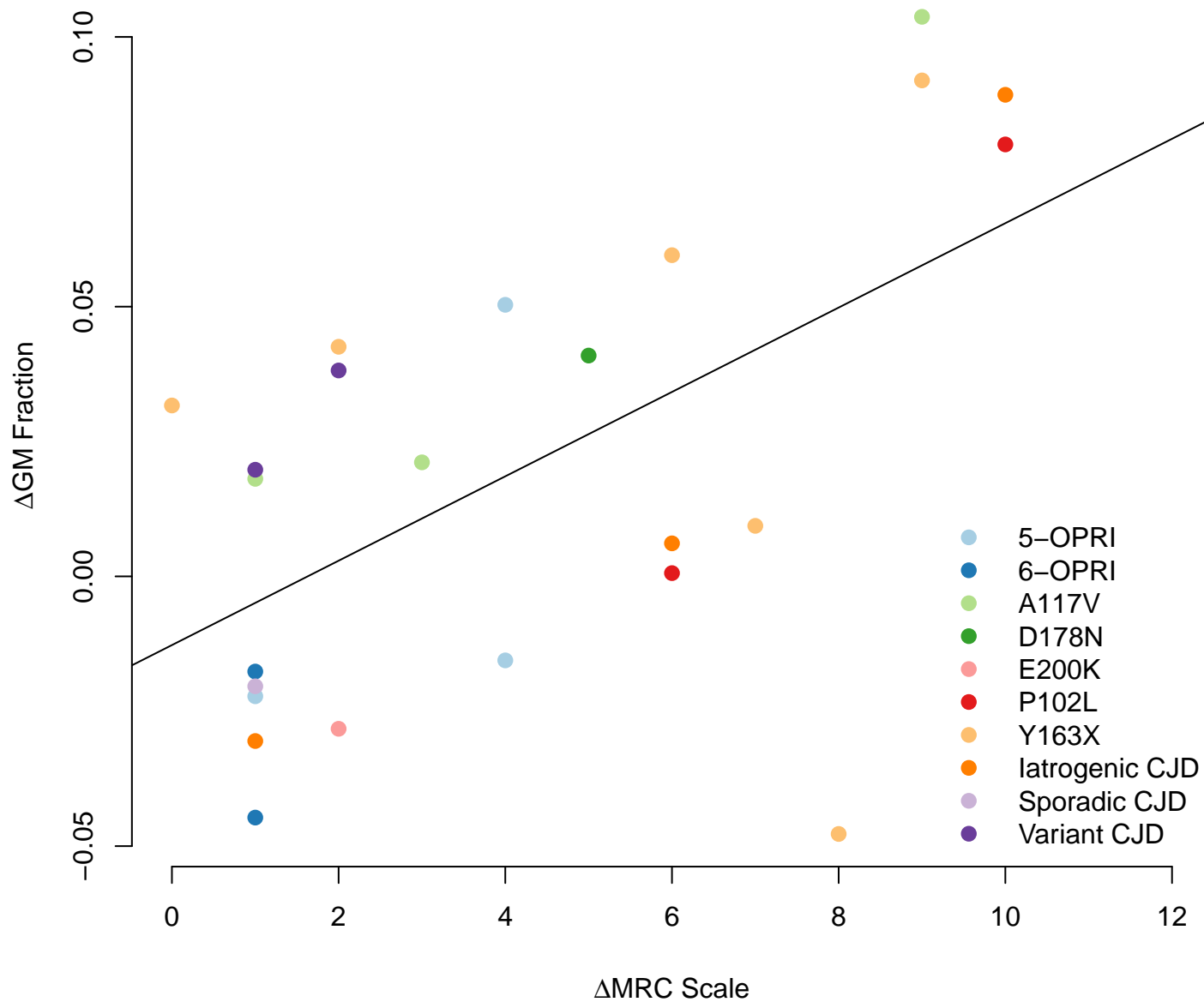

Supplement: Supplementary Fig. S4 — For each of the 24 patients, the variation in GM (ΔGM) is plotted vs the variation in MRC Scale (ΔMRC) between first and last scan for the right putamen (A) and the right head of caudate (B). Different etiologies or inherited mutations are shown in different colours as in Fig. 1. Linear regression equations and R(Ashburner and Friston, 2000) are: y = − 0.004719 + 0.006664 x, R(Ashburner and Friston, 2000) = 0.394 for the right putamen and y = − 0.01274 + 0.00782 x, R(Ashburner and Friston, 2000) = 0.342 for the right head of caudate. [file mmc4.pdf]
